# β2-microglobulin and colorectal cancer among inpatients: a case–control study

**DOI:** 10.1038/s41598-023-39162-x

**Published:** 2023-07-27

**Authors:** Huijie Wang, Huanwei Zheng, Xu Cao, Ping Meng, Jinli Liu, Caihua Zheng, Haiying Zuo, Zhichao Wang, Teng Zhang

**Affiliations:** 1 Department of Endoscopy, Shijiazhuang Traditional Chinese Medicine Hospital, Shijiazhuang, China; 2 Department of Gastroenterology, Shijiazhuang Traditional Chinese Medicine Hospital, Shijiazhuang, China; 3 Graduate School, Hebei North University, Zhangjiakou, China; 4 Institute of Traditional Chinese Medicine, North China University of Science and Technology, Tangshan, China

**Keywords:** Cancer, Gastrointestinal cancer, Risk factors

## Abstract

Β2-microglobulin (β2-M) is associated with various malignancies. However, the relationship between β2-M and colorectal cancer (CRC) remains unclear. We explored the association between β2-M and CRC among inpatients who underwent colonoscopy and explored factors that may modify the association. All consecutive inpatients who underwent colonoscopy were enrolled in a tertiary hospital between April 2015 and June 2022. Inpatients with initial CRC or normal colonoscopies were considered eligible as cases or controls, respectively. Baseline characteristics and laboratory indicators of the participants were collected from electronic medical records. Logistic regression analysis, smooth curve fitting, sensitivity analysis, and subgroup analysis were conducted in the present study. After adjusting for baseline clinical characteristics and laboratory parameters, β2-M was positively associated with CRC (odds ratio [OR] 1.32; 95% confidence interval [CI] 1.11–1.58) among inpatients. When the β2-M level was assigned as tertiles, participants in the highest tertile presented with a higher risk of CRC (OR 2.33; 95% CI 1.57–3.48). A positive linear association was observed between β2-M and CRC with smooth curve fitting. In particular, it may be of great importance to monitor β2-M levels for predicting CRC patients.

## Introduction

Colorectal cancer (CRC) is the second most fatal malignancy and third most common cancer^1^. The burden of CRC is increasing globally owing to its increasing prevalence. Studies have shown that CRC has the highest all-age incidence in China (607,900, 95% uncertainty interval [UI] 521,805–708,420])^2^. Although early diagnosis can significantly improve prognosis, patients with CRC often have no typical clinical presentation or present with only non-specific signs in the early stages, which leads to a low early diagnosis rate. The study of risk factors for CRC could open up further opportunities for the prevention, diagnosis, and treatment of CRC, which faces enormous challenges.

β2-microglobulin (β2-M), a well-known housekeeping protein, is non-glycosylated^3^. It not only has a tumor immunological role: it presents antigenic peptides to cytotoxic T lymphocytes, actively binding and lysing antigen-presenting cancer cells upon recognition of exogenous antigenic peptides on the cell surface^4–7^. β2-M also has both tumor-promoting and tumor-suppressing functions and is cancer cell background-dependent^8^. In addition, it has been shown that β2-M is widely implicated in the regulation of cancer cell growth, survival, apoptosis, and even metastasis^9,10^.

Under physiological conditions, β2-M exists at low levels in serum, urine, and other body fluids^11^. However, evidence has linked β2-M levels to various malignancies. Increased β2-M levels in body fluids (such as serum, plasma, or urine) are seen in patients with malignancies, including breast cancer, renal cell carcinoma, oral squamous cell carcinoma, lung cancer, gastrointestinal tract cancer, prostate cancer, lymphocytic malignancies, and multiple myeloma^12–17^, and can promote tumor growth through specific axes^18^. However, a previous study in 1980 showed no direct correlation between elevated β2-M levels and gastrointestinal cancer^19^.

Based on the fact that the relevance of β2-M to CRC is unclear, the association of β2-M with CRC still deserves to be studied. Our study was aimed to explore the association between β2-M and CRC in more different characteristics.

## Result

### Baseline characteristics

**Figure 1:**
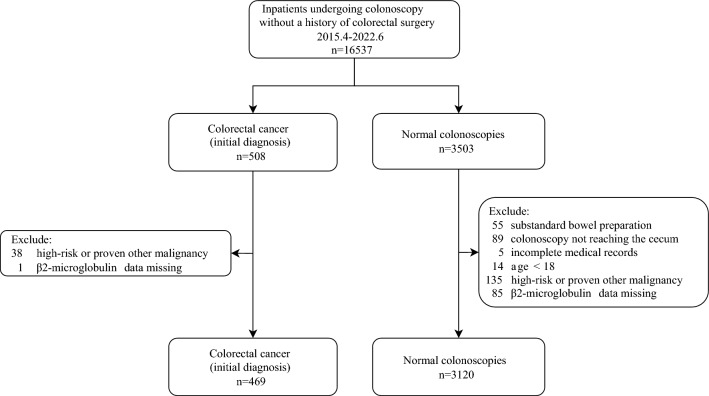
Flowchart of participants.

A total of 3589 consecutive inpatients were enrolled in this study between April 2015 and June 2022 (Fig. 1). The detailed characteristics of the individuals by case and control are presented in Table 1. CRC was associated with several variables such as age, sex, weight, smoking status, drinking status, β2-M, and several blood indicators.

**Table 1:** Baseline characteristics.

Variables	Case (n = 469)	Control (n = 3120)	*P* value
Age, year	64.8 ± 11.1	50.6 ± 12.8	**< 0.001**
Sex, male, n (%)	267 (56.9)	1176 (37.7)	**< 0.001**
Weight, kg	68.0 ± 11.1	66.5 ± 12.4	**0.018**
Marital status, n (%)			0.711
Single/ divorced	24 (5.1)	146 (4.7)	
Married	423 (90.2)	2802 (89.8)	
Others	22 (4.7)	172 (5.5)	
Smoking status, n (%)			**< 0.001**
Non-smoker	327 (69.7)	1997 (64)	
Current smoker	26 (5.5)	89 (2.9)	
Ex-smoker	3 (0.6)	21 (0.7)	
NA	113 (24.1)	1013 (32.5)	
Drinking status, n (%)			**0.014**
Non-drinker	328 (69.9)	1986 (63.7)	
Current drinker	20 (4.3)	123 (3.9)	
Ex-drinker	3 (0.6)	12 (0.4)	
NA	118 (25.2)	999 (32)	
Family history, n (%)			
Colorectal cancer	4 (0.9)	38 (1.2)	0.493
Digestive system malignancy	18 (3.8)	149 (4.8)	0.369
β2-M, mg/L	2.0 (1.6, 2.5)	1.5 (1.2, 1.7)	**< 0.001**
GFR, mL/min/1.73 m^2^	103.5 ± 21.5	95.0 ± 21.3	**0.001**
TG, mmol/L	1.3 (0.9, 1.7)	1.3 (0.9, 1.8)	0.692
TC, mmol/L	4.8 ± 1.0	5.0 ± 1.0	**< 0.001**
LDL, mmol/L	2.8 ± 0.7	2.9 ± 0.7	**0.011**
HDL, mmol/L	1.3 ± 0.3	1.4 ± 0.3	**< 0.001**
Apo A1, g/L	1.2 ± 0.3	1.3 ± 0.3	**< 0.001**
Lp(a), mg/L	198.0 (100.0, 381.0)	136.0 (77.0, 282.7)	**< 0.001**
Apo B, g/L	1.0 ± 0.2	1.0 ± 0.2	0.884
ALB, g/L	41.9 ± 4.3	44.5 ± 3.6	**< 0.001**
TP, g/L	69.7 ± 6.1	72.1 ± 5.2	**< 0.001**
ALT, U/L	14.0 (10.4, 19.0)	17.0 (12.9, 25.0)	**< 0.001**
AST, U/L	18.0 (15.0, 21.2)	19.3 (16.4, 24.0)	**< 0.001**
GGT, U/L	19.0 (14.0, 28.0)	18.0 (14.0, 27.0)	0.059
ALP, U/L	87.8 ± 45.1	73.8 ± 24.3	**< 0.001**
UA, μmol/L	301.9 ± 86.4	299.6 ± 94.2	0.612
GLU, mmol/L	6.8 ± 2.6	5.9 ± 1.7	**< 0.001**
CREA, μmol/L	69.6 ± 33.3	62.6 ± 16.7	**< 0.001**
Urea, mmol/L	5.1 ± 1.9	4.7 ± 1.5	**< 0.001**
5′-NT, U/L	3.0 (3.0, 4.0)	3.0 (3.0, 4.0)	0.67
ChE, U/L	7596.4 ± 1863.1	8894.1 ± 1945.6	**< 0.001**
TBA, μmol/L	3.0 (1.5, 5.0)	2.5 (1.4, 4.3)	**< 0.001**
DBIL, μmol/L	2.8 (2.0, 3.8)	2.7 (2.0, 3.7)	0.369
TBIL, μmol/L	11.5 (8.5, 14.9)	12.6 (9.8, 16.6)	**< 0.001**
Comorbidities, n (%)			
Hypertension	179 (38.2)	672 (21.5)	**< 0.001**
Ischemic cerebrovascular disease	57 (12.2)	295 (9.5)	0.067
CHD	61 (13)	329 (10.5)	0.11
HLP	18 (3.8)	294 (9.4)	**< 0.001**
Liver disease	30 (6.4)	347 (11.1)	**0.002**
DM	85 (18.1)	278 (8.9)	**< 0.001**
Kidney disease			0.465
CKD	12 (2.6)	58 (1.9)	
Others (kidney cysts and stones)	17 (3.6)	95 (3)	

Data are presented as the N (%), median (quartile 1–quartile 3), or mean ± SD. Bold values indicate statistical significance.

*β2-M* β2-microglobulin, *GFR* glomerular filtration rate, *TG* triglyceride, *TC* total cholesterol, *HDL* high-density lipoprotein, *LDL* low-density lipoprotein, *Apo A1* apoprotein A1, *Lp(a)* lipoprotein(a), *Apo B* apolipoprotein B, *ALB* albumin, *TP* total protein, *ALT* alanine aminotransferase, *GGT* gamma-glutamyl transferase, *ALP* alkaline phosphatase, *AST* aspartate aminotransferase, *UA* uric acid, *GLU* glucose, *CREA* creatinine, *ChE* cholinesterase, *5′-NT* 5′-nucleotidase, *TBA* total bile acid, *DBIL* direct bilirubin, *TBIL* total bilirubin, *CHD* coronary heart disease, *HLP* hyperlipidemia, *DM* diabetes mellitus, *CKD* chronic kidney disease, *NA* not recorded.

### Relationship between β2-microglobulin and colorectal cancer

**Figure 2:**
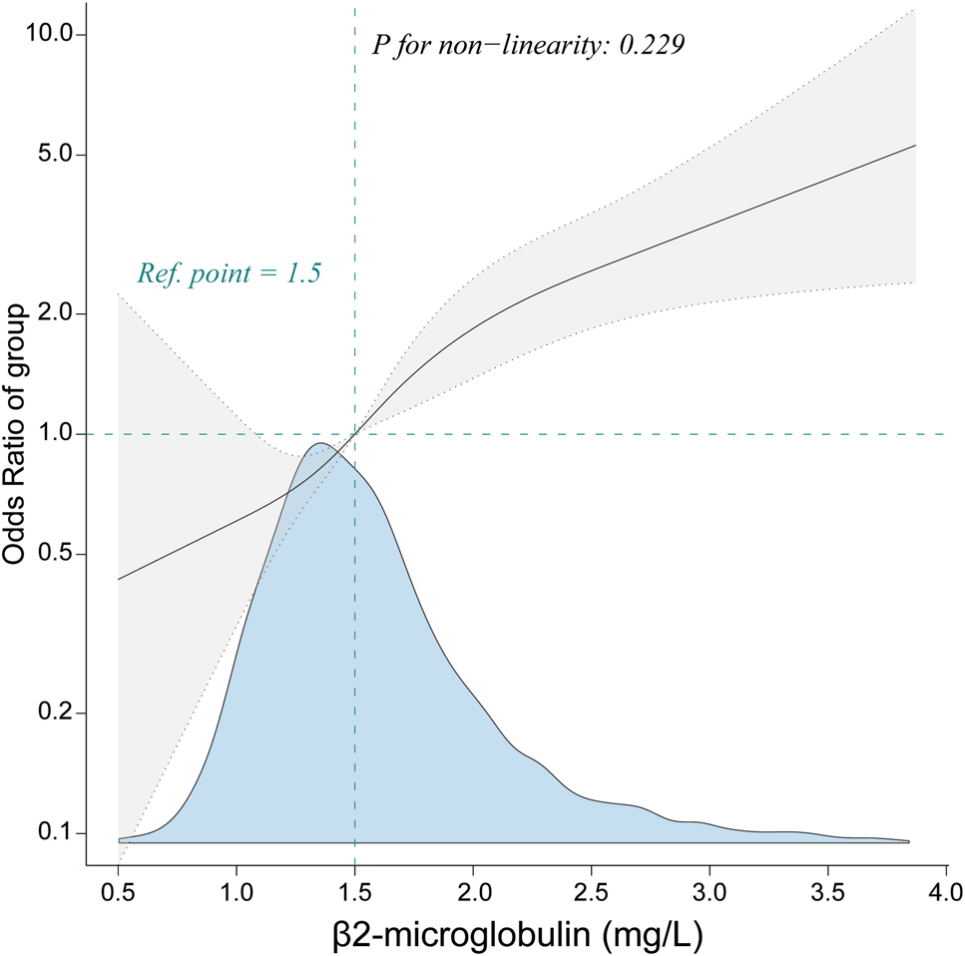
Linear relationship between β2-microglobulin and colorectal cancer among inpatients (Only 99% of the data is shown). Adjustment by sex, age, weight, drinking status, smoking status, glomerular filtration rate, total cholesterol, apoprotein A1, lipoprotein(a), alanine aminotransferase, albumin, total protein, aspartate aminotransferase, alkaline phosphatase, glucose, urea, cholinesterase, total bilirubin, total bile acid, hyperlipidemia, hypertension, diabetes mellitus, and liver disease.

We performed smooth curve fitting and found a positive linear association between β2-M and the risk of CRC (*P* for non-linearity: 0.229, only 99% of data is shown in Fig. 2).

When β2-M was evaluated as a continuous variable, multivariable-adjusted regression analysis (adjustment for age, sex, weight, drinking status, smoking status, glomerular filtration rate [GFR], total cholesterol [TC], apoprotein A1 [Apo A1], lipoprotein(a) [Lp(a)], total protein [TP], albumin [ALB], alanine aminotransferase [ALT], aspartate aminotransferase [AST], alkaline phosphatase [ALP], glucose [GLU], urea, total bile acid [TBA], cholinesterase [ChE], total bilirubin [TBIL], diabetes mellitus [DM], liver disease, hypertension, and hyperlipidemia [HLP]) demonstrated that there was a 32% higher risk of CRC with each 1 mg/L increment in β2-M level (Table 2, Model II, OR 1.32; 95% CI 1.11–1.58]). When β2-M was analyzed as tertiles, also in a fully adjusted model (Table 2, Model II, *P* for trend was < 0.001), the adjusted OR for CRC in tertile 2 and tertile 3 were 1.35 (95% CI 0.90–2.02) and 2.33 (95% CI 1.57–3.48), respectively, with tertile 1 as reference. This means that the risk of CRC increased with increasing β2-M levels.

**Table 2:** Multivariable logistic regression analyses of β2-microglobulin and colorectal cancer.

Variable	Event, n (%)	Unadjusted Model^a^		Model I^b^		Model II^c^	
		OR (95% CI)	*P* value	OR (95% CI)	*P* value	OR (95% CI)	*P* value
β2-M, mg/L	469/3589 (13.1)	3.05 (2.61–3.57)	< 0.001	1.55 (1.32–1.81)	< 0.001	1.32 (1.11–1.58)	0.002
β2-M tertile, mg/L							
< 1.34	43/1166 (3.7)	1 (Reference)		1 (Reference)		1 (Reference)	
1.34–1.69	99/1190 (8.3)	2.37 (1.64–3.42)	< 0.001	1.35 (0.92–1.98)	0.127	1.35 (0.9–2.02)	0.149
≥ 1.70	327/1233 (26.5)	9.43 (6.78–13.11)	< 0.001	3.09 (2.15–4.42)	< 0.001	2.33 (1.57–3.48)	< 0.001
*P* for trend			< 0.001		< 0.001		< 0.001

*OR* odds ratio, *CI* confidence interval, *β2-M* β2-microglobulin, *GFR* glomerular filtration rate, *TC* total cholesterol, *Apo A1* apoprotein A1, *Lp(a)* lipoprotein(a), *ALT* alanine aminotransferase, *ALB* albumin, *TP* total protein, *ALP* alkaline phosphatase, *AST* aspartate aminotransferase, *GLU* glucose, *ChE* cholinesterase, *TBIL* total bilirubin, *TBA* total bile acid, *HLP* hyperlipidemia, *DM* diabetes mellitus.

^a^No adjustment.

^b^Adjusted for age and sex.

^c^Adjusted for Model I + weight, drinking status, smoking status, GFR, TC, Apo A1, Lp(a), ALT, ALB, TP, ALP, AST, GLU, urea, ChE, TBIL, TBA, HLP, hypertension, liver disease, and DM.

### Sensitivity analysis

**Figure 3:**
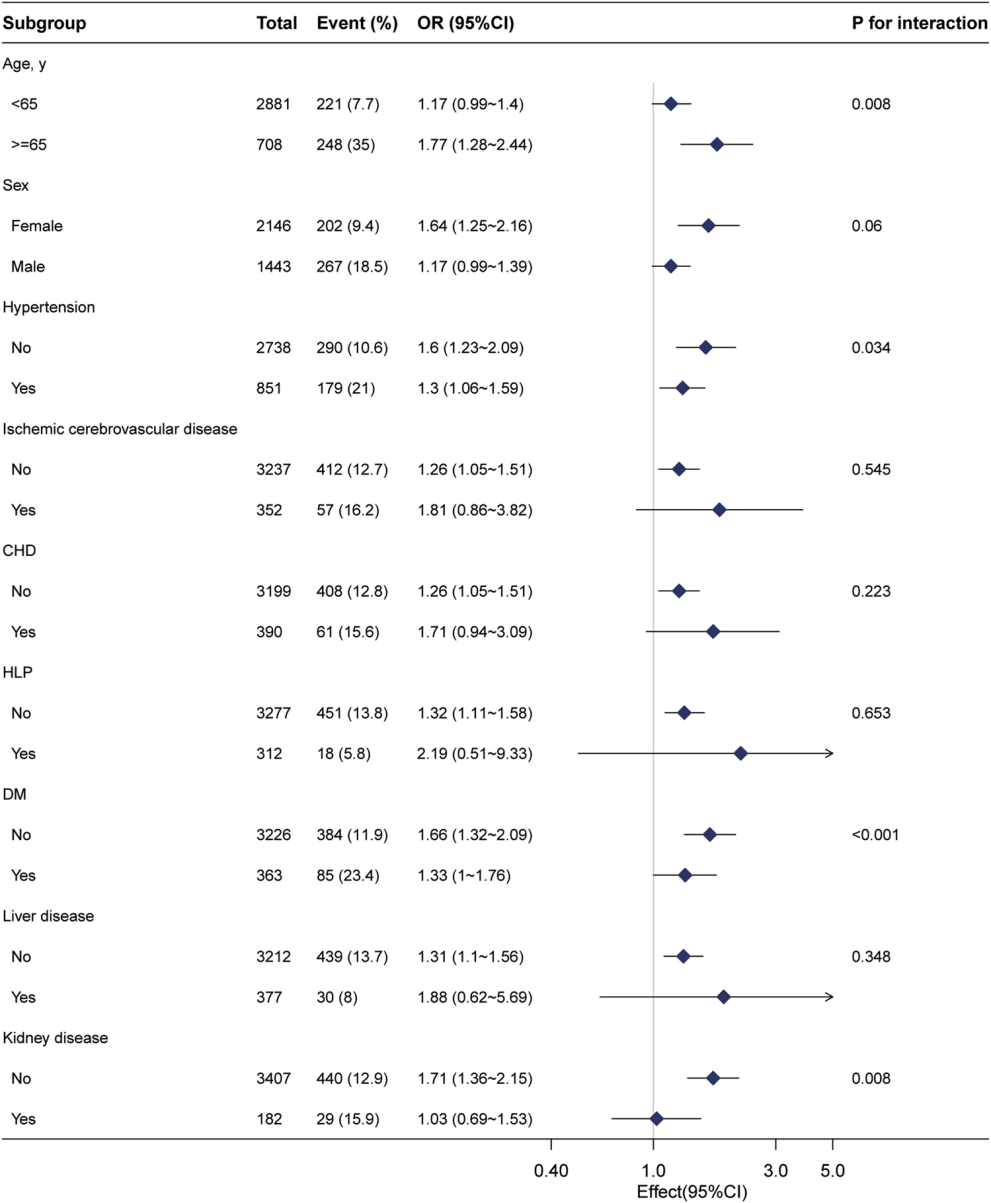
Subgroup analyses of the β2-microglobulin and colorectal cancer among inpatients. Adjusted for sex, age, weight, drinking status, smoking status, glomerular filtration rate, total cholesterol, apoprotein A1, lipoprotein(a), alanine aminotransferase, albumin, total protein, aspartate aminotransferase, alkaline phosphatase, glucose, urea, cholinesterase, total bilirubin, total bile acid, hyperlipidemia, hypertension, liver disease, and diabetes mellitus.

Subgroup analysis (Fig. 3) was conducted to investigate the effect of β2-M on CRC in different subgroups. The relationship between β2-M and CRC was adjusted for the following subgroups: age, sex, HLP, hypertension, liver disease, ischemic cerebrovascular disease, coronary heart disease (CHD), kidney disease, and DM. *P* values < 0.05 for interactions in age, sex, and kidney disease subgroups may not be statistically significant based on multiple testing. However, in the DM subgroup, a stronger association between β2-M and CRC was observed among inpatients without DM.

After the exclusion participants with missing data, there were 2210 participants and the association between β2-M and CRC remained robust in the multivariable logistic regression models (Supplementary Table S1). When β2-M was analyzed as a continuous variable, multivariable-adjusted regression analysis demonstrated (adjustment for age, sex, weight, drinking status, smoking status, GFR, TC, Apo A1, Lp(a), ALT, ALB, TP, ALP, AST, GLU, urea, ChE, TBIL, TBA, HLP, hypertension, liver disease, and DM) that there was a 19% higher risk of CRC with each 1 mg/L increment in β2-M level (Model II, OR 1.19 [95% CI 1.01–1.4]). When β2-M was analyzed as tertiles, also in a fully adjusted model (Model II, *P* for trend was < 0.001), with tertile 1 as reference, the adjusted OR for CRC in tertile 2 and tertile 3 were 1.22 (95% CI 0.73–2.04) and 2.73 (95% CI 1.65–4.5), respectively.

In addition, propensity score matching (PSM) indicated that the relationship between β2-M and CRC was robust (Supplementary Table S2).

## Discussion

The present study revealed a positive linear relationship between β2-M and CRC among inpatients in China.

The relationship between β2-M and CRC has been previously examined but is inconclusive. An earlier study reported no direct relationship between β2-M and gastrointestinal cancer^19^. This finding is inconsistent with ours, probably because of differences in cancer populations and sample sizes. Furthermore, in a cohort study, a 121% increased risk of colorectal cancer (n = 255) was found in participants in the highest and lowest quartiles of serum β2-M concentration^16^. This finding was consistent with the present study. In addition, this study found a positive linear association between β2-M and colorectal cancer. This may be due to region and sample size.

In this study, we considered the possibility that unknown confounding factors might influence the relationship between β2-M and colorectal cancer. Therefore, we collected all available clinical factors and adjusted for additional confounding factors to exclude such effects. Our finding that DM modified the association between β2-M and CRC among inpatients requires further exploration. As shown in Fig. 3, a stronger positive relationship was observed in individuals without DM, which requires further exploration.

β2-M is an interesting protein with multiple biological functions that are closely associated with cancer. Under normal conditions, β2-M acts as a friend to humans, forming complexes with the heavy chain of major histocompatibility complex class I molecules (MHC-I) in a well-known manner, presenting antigenic peptides to cytotoxic T cells, thereby allowing T cells to recognize foreign peptide antigens on the cell surface to destroy tumor cells and act as immune surveillance agents^5,20–22^. However, in the early stages of cancer, tumor cells exhibit impaired β2-M expression, resulting in defective antigen processing and presentation, thereby evading immune surveillance^23^. In cancer patients, β2-M acts as an enemy in humans and as an oncogenic factor that stimulates the growth and progression of cancers^24–26^, in particular, β2-M acts directly on tumor cells to increase their growth, survival, and invasiveness by inducing epithelial-to-mesenchymal transition^26^. In an animal study, Josson et al. showed that β2-M overexpression promoted the growth and progression of human lung, breast, prostate, and kidney tumor cells, leading to metastatic and lethal findings^27^. However, there were no participants with colorectal cancer in these studies, and further research is needed.

Under normal physiological conditions, β2-M exists at low levels in human fluids. This is because the cell-associated form of β2-M is not anchored to the cell membrane, allowing dissociation and balanced exchange with soluble β2-M circulating in the extracellular fluid. β2-M is metabolized in the glomerulus after its dissociation from the MHC-I heavy chain to maintain stability in humans^28,29^. Although elevated serum β2-M levels suggest an abnormal condition seen in a variety of malignancies^12,15,17,25,30,31^, our study adds to the evidence on colorectal cancer that β2-M may serve as a potential biomarker for colorectal cancer^18^. Furthermore, there is considerable evidence that colorectal cancer is associated with immune response and inflammation^32–35^, and we believe that β2-M probably plays a role in early CRC.

There are two possible explanations for the elevated serum β2-M in patients with colorectal cancer: first, the assembly of β2-M and class I heavy chain on the tumor cell membrane does not result from conformational changes in the heavy chains, leading to reduced expression of class I antigen on tumor cells and the accumulation of free β2-M in the serum; secondly, there is increased renewal of tumor cells in patients with colorectal cancer, resulting in increased shedding of β2-M from tumor cells and subsequently elevated serum levels.

This study had some limitations, such as missing data, due to the retrospective nature. However, the sensitivity and subgroup analyses showed that the results were robust. In addition, this study is not representative of the relationship between long-term β2-M levels and CRC because of the lack of data on repeated measurements of β2-M. Furthermore, the participants were from a tertiary hospital in China, which may limit their generalizability to other populations. And the effects of variables not available in this study cannot be excluded. Finally, Longitudinal studies are needed to confirm the causal relationship between β2-M and CRC.

In conclusion, our study indicated a positive linear association between β2-M and CRC in inpatients. Further studies are needed to investigate the potential mechanism of β2-M on CRC risk. β2-M may be used as a biomarker for the diagnosis and management of CRC in the future.

## Materials and methods

### Population

All consecutive inpatients who underwent colonoscopy were enrolled in a tertiary hospital between April 2015 and June 2022 in the current study. Inpatients with initial CRC or normal colonoscopy were considered eligible case (n = 469) or control (n = 3120) groups, respectively. Flowchart presents the screening of participants (Fig. 1), including the reasons for exclusion. Participants were included only once.

### Ethics approval

This retrospective study was approved by the Ethics Review Board of Shijiazhuang Traditional Chinese Medicine Hospital (No.20220919029) and the requirement for informed consent was waived because of the retrospective nature of the study. All methods were performed in accordance with the relevant guidelines and regulations.

### Covariates

Baseline characteristics and laboratory indicators were collected from electronic medical records. The data analyzed in this study are listed in Table 1, including demographic data, comorbidities (including hypertension, ischemic cerebrovascular disease, CHD, HLP, liver disease, kidney disease, and DM), and laboratory indicators (including β2-M, GFR, triglyceride [TG], TC, low-density lipoprotein (LDL), high-density lipoprotein (HDL), Apo A1, Lp(a), apolipoprotein B [Apo B], ALB, TP, ALT, gamma-glutamyl transferase [GGT]; ALP, AST, GLU, CREA, urea, uric acid [UA], ChE, 5′-nucleotidase [5′-NT], TBA, direct bilirubin [DBIL], and TBIL). Drinking and smoking statuses were divided into four groups: never, former, current, and NA (not recorded). Liver disease included cirrhosis, fatty liver disease, and hepatitis. Kidney disease included chronic kidney disease [CKD], kidney cysts, and kidney stones. GFR was calculated using the modification of diet in renal disease equation^36^. Laboratory data were the first eligible results obtained during hospitalization (before colonoscopy).

### Statistical analysis

The participants’ characteristics were assessed according to the following groups: CRC and control groups. Depending on the nature of the variables, we used the Chi-square test, Fisher's exact test, Mann–Whitney *U* test, or Student's *t* test, respectively.

We considered β2-M as a continuous variable in the primary analysis, calculated the odds ratios (ORs) per 1 standard deviation (SD) increment, and presented *P* for trend. We also classified β2-M into tertiles and calculated ORs using the lowest tertile as the reference. Three logistic regression models were used. The unadjusted model had no adjustment for covariates. The minimally adjusted model only included sex and age. The fully adjusted model further included weight, drinking status, smoking status, GFR, TC, Apo A1, Lp(a), ALT, ALB, TP, ALP, AST, GLU, urea, ChE, TBIL, TBA, HLP, hypertension, liver disease, and DM to account for the potential confounding by β2-M and CRC. These potential confounders were screened according to a change in the effect estimate ≥ 10% and significant covariates in the univariate analysis (*P* < 0.05). We used the multiple imputation method for the covariates with missing data. Smooth curve fitting was performed to evaluate the relationship between β2-M levels and CRC. Additionally, participants were matched for the fully adjusted model using a one-to-one nearest neighbor technique with a caliper width of 0.2 in PSM. ORs and 95% confidence intervals (95% CIs) for the association between β2-M and CRC were calculated using the logistic regression model.

Furthermore, potential modifications of the relationship between β2-M and CRC were assessed, including the following subgroups: sex, age (< 65 vs. ≥ 65 years), HLP, hypertension, liver disease, kidney disease, ischemic cerebrovascular disease, CHD, and DM. Subgroup heterogeneity was assessed by multivariate logistic regression and subgroup interactions by likelihood ratio testing.

A 2-tailed *P* < 0.05 was recognized as statistically significant. All analyses were conducted using the statistical software packages R 3.3.2 (http://www.R-project.org, the R Foundation) and Free Statistics software version 1.7.1.

## Supplementary Information

Supplementary Table S1.

Supplementary Table S2.

## Data Availability

The datasets analyzed during the current study are available from the corresponding author on reasonable request.
