# Supplementary material for: β2-microglobulin and colorectal cancer among inpatients: a case–control study
Source: Sci Rep. 2023 Jul 27;13:12222. doi: 10.1038/s41598-023-39162-x (PMC10374627; doi:10.1038/s41598-023-39162-x)
Supplement: Supplementary file 1 — Supplementary Table S1. [file 41598_2023_39162_MOESM1_ESM.docx]

Table S1 Multivariable logistic regression analyses of β2-microglobulin and colorectal cancer.

| Variable | Event, n (%) | Crude Model | |  | Model I | |  | Model II | |
| --- | --- | --- | --- | --- | --- | --- | --- | --- | --- |
|  |  | OR (95% CI) | *P* value |  | OR (95% CI) | *P* value |  | OR (95% CI) | *P* value |
| β2-M, mg/L | 309/2210 (14) | 2.81 (2.33~3.39) | <0.001 |  | 1.49 (1.24~1.8) | <0.001 |  | 1.19 (1.01~1.4) | 0.04 |
| β2-M tertile, mg/L | |  |  |  |  |  |  |  |  |
| <1.35 | 29/733 (4) | 1(Reference) |  |  | 1(Reference) |  |  | 1(Reference) |  |
| 1.35-1.71 | 61/738 (8.3) | 2.19 (1.39~3.45) | 0.001 |  | 1.21 (0.75~1.96) | 0.431 |  | 1.22 (0.73~2.04) | 0.438 |
| ≥1.72 | 219/739 (29.6) | 10.22 (6.83~15.31) | <0.001 |  | 3.43 (2.2~5.34) | <0.001 |  | 2.73 (1.65~4.5) | <0.001 |
| *P* for trend |  |  | <0.001 |  |  | <0.001 |  |  | <0.001 |

Abbreviations: OR, odds ratio; CI, confidence interval; β2-MG, β2-microglobulin; GFR, glomerular filtration rate; TC, total cholesterol; Apo A1, apoprotein A1; HDL, high-density lipoprotein; LDL, low-density lipoprotein; Lp(a), lipoprotein(a); ALT, alanine aminotransferase; ALB, albumin; TP, total protein; GGT, γ-glutamyl transferase; ALP, alkaline phosphatase; AST, aspartate aminotransferase; GLU, glucose; CREA, creatinine; ChE, cholinesterase; TBIL, total bilirubin; TBA, total bile acid; HLP, hyperlipemia; DM, diabetes mellitus.

Crude model: no other covariates were adjusted.

Model I: adjusted for sex and age.

Model II: adjusted for Model I + weight, drinking status, smoking status, GFR, TC, Apo A1, Lp(a), ALT, ALB, TP, GGT, ALP, AST, GLU, urea, ChE, TBIL, TBA, HLP, hypertension, liver disease, and DM.
