# Supplementary material for: β2-microglobulin and colorectal cancer among inpatients: a case–control study
Source: Sci Rep. 2023 Jul 27;13:12222. doi: 10.1038/s41598-023-39162-x (PMC10374627; doi:10.1038/s41598-023-39162-x)
Supplement: Supplementary file 2 — Supplementary Table S2. [file 41598_2023_39162_MOESM2_ESM.docx]

**Table** **S2** Associations between β2-microglobulin and colorectal cancer in the crude analysis, multivariable analysis, and propensity score matching analysis.

| Analysis | OR (95% CI) | *P* value |
| --- | --- | --- |
| Crude analysis^a^ | 3.05 (2.61~3.57) | <0.001 |
| Multivariable adjusted analysis^b^ | 1.32 (1.11~1.58) | 0.002 |
| Propensity score matched^c^ | 1.43 (1.15~1.77) | 0.001 |

Abbreviations: CI, confidence interval; OR, odds ratio; GFR, glomerular filtration rate; TC, total cholesterol; Apo A1, apoprotein A1; lipoprotein(a) (Lp[a]), ALT, alanine aminotransferase; ALB, albumin; TP, total protein; ALP, alkaline phosphatase; AST, aspartate aminotransferase; GLU, glucose; ChE, cholinesterase; TBIL, total bilirubin; TBA, total bile acid; HLP, hyperlipidemia; DM, diabetes mellitus; CRC, colorectal cancer.

^a^Shown is the OR from the logistic regression model without adjusting any covariates.

^b^Shown is the OR from the multivariable logistic regression model adjusted for Model II (sex, age, weight, drinking status, smoking status, GFR, TC, Apo A1, Lp(a), ALT, ALB, TP, ALP, AST, GLU, urea, ChE, TBIL, TBA, HLP, hypertension, liver disease, and DM).

^c^Shown is the OR from a logistic regression model after propensity score matching (matched for Model II). The analysis included 744 participants (372 CRCs and 372 controls).
